# Pediatric hereditary angioedema: an update

**DOI:** 10.12688/f1000research.11320.1

**Published:** 2017-07-24

**Authors:** Geetika Sabharwal, Timothy Craig

**Affiliations:** 1 Division of Pulmonary, Allergy and Critical Care, Department of Allergy and Immunology, Penn State University, Milton S. Hershey Medical Center, Hershey, PA, USA

**Keywords:** Hereditary angioedema, Genetic disorder, C1-INH gene, HAE

## Abstract

Hereditary angioedema (HAE) with C1-inhibitor (C1-Inh) deficiency (C1-Inh-HAE) is a rare, life-threatening, and disabling genetic disorder characterized by self-limited tissue swelling caused by deficiency or dysfunction of C1-Inh. Our aim in this update is to discuss new advances in HAE therapy, focusing mainly on the various treatment options that have become available recently and also drugs that are under trial for prophylaxis to prevent attacks. There is a paradigm shift to where the treatment of HAE is headed, focusing now on prophylactic treatment rather than abortive management.

## Introduction

Hereditary angioedema (HAE) with C1-inhibitor (C1-Inh) deficiency (C1-Inh-HAE) is a rare, life-threatening, and disabling genetic disorder characterized by self-limited tissue swelling that most often affects the skin, upper respiratory tract, and gastrointestinal tract caused by deficiency or dysfunction of the *C1INH* gene ( *SERPING1* gene), which was mapped to chromosome 11 (11q12-q13.1). C1-Inh is a serpin-regulating complement system, intrinsic coagulation system to a degree, fibrinolytic system, and, as a strategic target, the contact system (Hageman factor and plasma kallikrein) ^1, 2^. The estimated prevalence of C1-Inh-HAE is 1 in 50,000, with reported ranges from 1:10,000 to 1:150,000 ^1, 3– 5^. C1-Inh-HAE has been reported in both males and females of all races ^6^. In a large series of C1-Inh-HAE patients reported, the illness had a mean age of onset at 11.2 years ^7^, with almost 90% of patients experiencing onset of symptoms by age 20 ^7, 8^. A child is expected to have a 50% chance of inheriting the disease from either parent with the mutation; however, about 20–25% of mutations occur spontaneously in patients and thus without a family history.

Bradykinin is considered to be a responsible mediator of angioedema upon interaction with B _2_ receptor on the endothelium. A local activation process at the site of the angioedema attack cannot explain a pathogenic model and support recorded observations after treatment application. A recent model for angioedema attacks in HAE patients has been proposed, with a systemic, fluid-phase activation of the contact system to generate bradykinin and its first metabolite ^9^; both subsequently interact with endothelial receptors that are locally expressed in the affected tissues rather than with constitutively expressed receptors. This makes B _1_ receptor strategically important, a situation pertaining to endothelium induction upon inflammatory stimuli, often recognized as triggers of angioedema attacks.

## Classification

As established at the gene level, three types of HAE have been described. C1-Inh-HAE is the prototypical example of kinin-dependent HAE, with type 1 and type 2. Type 1 is characterized by low production of functionally active C1-Inh and accounts for 80–85% of cases. Type 2 HAE is characterized by normal or elevated levels of C1-Inh but with functional impairment of the protein and accounts for 15–20% of cases. Recently, a type 3 HAE has been provisionally described. Type 3 presents with similar clinical manifestations to the first two types, but its biological phenotype differs in that there are no abnormalities in C1-Inh level or function. Associated with a gain-of-function, mutations in coagulation factor XII protease (Hageman factor) with dominant inheritance are observed to occur in factor XII-HAE cases (FXII-HAE), but the pathophysiology in the majority of HAE with normal C1-Inh has not been documented ^10– 15^.

## Assessing disease severity and impact on quality of life

The swelling caused by HAE is the primary symptom affecting an individual’s life, causing significant disabilities. Generic tools to assess quality of life for any chronic disease do not quantify the impact that angioedema has on one’s life. Newer tools like the Angioedema Quality of Life Questionnaire (AE-QoL) are valid and reliable for angioedema symptoms, and it can be used in both histamine- and bradykinin-mediated angioedema ^16^.

The AE-QoL consists of 17 questions from four domains including functioning, fears/shame, fatigue/mood, and food which have five answers each, and the patient has to answer for the past 4 weeks. The scores are then transformed to a linear 0–100 scale. Higher scores represent higher impairment of quality of life ^16^. The AE-QoL is also available in several languages (American English, Canadian English, Canadian French, Danish, Greek, Hungarian, Italian, Japanese, Mexican-Spanish, Dutch, French, German, and many more) and is free to use ^17, 18^.

HAE poses a considerable burden on patients and their families in terms of direct medical costs and indirect costs related to lost productivity. This burden is substantial at the time of and in between attacks ^19^.

## Present therapy

The management of pediatric C1-Inh-HAE is classified into three major categories, including treatment of acute attacks or on-demand therapy, short-term prophylaxis (pre-procedural), and long-term prophylaxis ^6, 20, 21^. Targeting one of the underlying defective pathways described above makes the recently introduced therapies effective.

### Agents available for on-demand therapy

Berinert is a C1-Inh (human) plasma-derived preparation which was approved in 2016 by the FDA to be used in children of all ages for treating acute abdominal, facial, or laryngeal C1-Inh-HAE attacks. It is delivered intravenously and is approved for on-demand treatment through self-administration or by a healthcare provider. It is used in a dose of 20 units/kg at a rate of 4 ml/minute, which helps adjust dosing for children of all weights. It was approved initially in 2009 in the US for adults and children older than 12 years of age but has been available in Europe for decades for attacks ^22^. Berinert was also found to be the most cost-effective drug compared to Firazyr or Kalbitor in patients weighing up to 330 lb ^23^.

Cinryze, also a plasma-derived and nanofiltered C1-Inh concentrate, is approved in Europe for attacks but is not yet approved in the US for attacks. It is used off label for attacks but approved for prophylaxis at a dose of 1,000 units twice a week; it has also been used in younger children but is not approved by the FDA for children younger than 13 years of age ^24, 25^.

Ruconest is a C1-Inh recombinant preparation which has been FDA-approved for treating acute C1-Inh-HAE attacks in children over 13 years of age ^26^. It is administered intravenously and is approved for self-administration. The required dose is 50 units/kg up to a maximum of 4,200 units ^6^. Similarly to Berinert, the dose is adjusted for weight, which makes it easy to adjust the dose for children. It was approved in the US in 2014 and in Europe in 2010 for the treatment of attacks ^6^.

Ecallantide (Kalbitor) is a plasma kallikrein inhibitor, a polypeptide that was developed from a *Kunitz* domain through phage display to mimic antibodies inhibiting kallikrein. It has been FDA approved to treat HAE attacks in patients 12 years of age and older. It is administered through subcutaneous injections. Due to a 3% risk of anaphylaxis, it is required to be given by a healthcare provider, either at home or in a healthcare setting. It is used at a dose of 30 mg subcutaneously and repeated within 24 hours if there is no improvement. It was approved initially in 2009 in the US. It is not approved in Europe. A recent analysis of pooled data suggests that ecallantide is effective for the treatment of HAE attacks in younger patients and has an acceptable safety profile, but it is not yet approved for children younger than 12 years of age ^27, 28^.

Icatibant (Firazyr) is a bradykinin B _2_ receptor antagonist, with clinical efficacy pointing to involvement of B _2_ receptor rather than B _1_ receptor. However, its affinity for B _1_ receptor is still higher than that of *des*Arg ^9^-bradykinin for B _1_ receptor ^29^. It is currently approved for the treatment of C1-Inh-HAE attacks in patients older than 18 years of age. It is approved only in Europe to treat pediatric patients. A pharmacokinetic, tolerability, and safety study of icatibant in patients younger than 18 years of age with HAE has been completed, demonstrating its safety and efficacy in children ^30^. The required dose of icatibant is 30 mg subcutaneously, in a large excess over the ligand to antagonize.

### Long-term prophylaxis

Pre-pubertal children usually have less severe disease, so they are often managed by on-demand therapy alone. But if they need long-term prophylaxis for recurrent, severe attacks, if they have co-morbid conditions, or if the family prefers, the following options are available ^30^. Patients on long-term prophylaxis should also have on-demand therapy available to them for breakthrough attacks.

***C1-inhibitor.*** Plasma-derived C1-Inh, like Berinert and Cinryze, can be used for prophylaxis. They have proven efficacy in clinical trials and practice and their long half-life enables administration every 3 to 4 days. Cinryze is used intravenously at a dose of 1,000 units twice weekly, but the dose can be increased to the desired effect, and is the only FDA-approved drug for prophylaxis. Berinert is used off label, but it has the advantage of a dose that is weight adjusted.

***Androgens.*** Anabolic androgens (synthetic 17-alpha-alkylated androgens) like danazol, stanozolol, oxandrolone, oxymetholone, tibolone, and methyltestosterone can be used for long-term prophylaxis, but danazol is more widely available ^31– 35^. The usual dose ranges from 50 to 200 mg of danazol, either daily or every other day. It is recommended to start with a 200 mg dose daily and then taper to the least-effective dose. Less often recommended is starting with a low dose and gradually increasing to effect. These agents cause dose-related side effects over time and significant side effects in children and therefore are not recommended for use in children in the United States ^36^.

Anti-fibrinolytics ε-aminocaproic acid (Amicar [EACA]) and a cyclic derivative tranexamic acid (TXA) can be used for prophylaxis ^14, 37, 38^. TXA is easier to administer and is better tolerated. Anti-fibrinolytics are preferred over androgens in children. They can be administered orally at a dose of 25 mg/kg daily up to 3 g/day, and if this dose does not help in a few weeks it is recommended to stop it. TXA has been used in Europe and Asia for decades. In the US, EACA has been available for many years, and an oral preparation of TXA became available in 2009 when it was approved by the FDA for the treatment of menorrhagia. The mechanism of action is poorly understood and is assumed to compromise molecular interactions in the plasminogen activation process, with a subsequent decrease of factor XII activation in promoting bradykinin generation. Anti-fibrinolytics are used off label for long-term prophylaxis but are minimally effective, and most experts consider them to be ineffective for the treatment of HAE attacks.

### Short-term prophylaxis/pre-procedural

Berinert or Cinryze, both of which are plasma-derived C1-Inh preparations, are used off label since no therapies are approved for this purpose in the USA.

Attenuated androgens are effective and can be administered at high doses (200 mg three times a day) for at least 5 to 7 days prior to the procedure and a few days after. They should be avoided in children unless other therapies are not available; however, short courses seem to be tolerated well with minimal adverse events and can even be used in children safely. Androgens should be avoided in women who are pregnant and lactating.

Fresh frozen plasma (FFP) is reported to prevent HAE attacks when administered before dental, medical, or surgical procedures, but controlled studies are lacking in children. In addition, the risk of allo-sensitization and anaphylaxis is greater with FFP than with C1-Inh preparations. It can be used at a dose of 1–2 units per patient.

Anti-fibrinolytics, EACA or TXA, are used off label for short-term or perioperative prophylaxis, but evidence of efficacy does not exist.

## Future of HAE treatment

Most investigation presently is focused on long-term prophylaxis ( Table 1). A phase 3 study to assess the safety and efficacy of subcutaneous administration of plasma-derived C1-Inh (CSL 830) was recently reported and is showing promising results. This study evaluated type I and type II C1-Inh-HAE patients during two 16-week treatment periods. The study assessed the number of attacks experienced and the number of times rescue medication was needed while receiving CSL 830 prophylactically and both were found to be significantly reduced. HAE attack rates were reduced by a median of 89% and 95% (for the 40 IU/kg and 60 IU/kg dose, respectively). Additionally, 40% of patients on the higher dose were completely free of attacks, and patients, in general, experienced fewer and milder angioedema symptoms. None of the patients on 60 IU/kg experienced a laryngeal attack within the study period. This drug is currently being proposed to the FDA and is likely to be approved soon for long-term prophylaxis (funded by CSL Behring; NCT01912456) ^39, 40^.

**Table 1.** Newer prophylactic therapies under trial for hereditary angioedema.

Products under investigation	CSL 830	BCX7353	DX-2930 (lanadelumab)	Ruconest
**Mechanism of action**	C1-inhibitor	Kallikrein inhibitor	Human monoclonal antibody against plasma kallikrein	Recombinant C1-inhibitor
**Phase of study**	3	2	1b	2
**Percent reduction of** **attacks (%)**	89% (40 IU/kg) 95% (60 IU/kg)	88% (peripheral) 24% * (abdominal)	100% (300 mg) 90% (400 mg)	72% (two/week) 44% (one/week)
**Mode of administration**	Subcutaneous	Oral	Subcutaneous	Intravenous
**Dose**	40–60 IU/kg	350 mg daily	300–400 mg	50 IU/kg

IU, international unit *drug gastrointestinal adverse effects may explain the difference in attack suppression between abdominal and other attacks.

Another similar study assessing the efficacy and safety of subcutaneous C1-Inh is in phase 3. The dose is only twice that of the intravenous preparation and thus the anticipated decrease in attacks is expected to be similar to the 1,000 units intravenously, which is about a 50% reduction of attacks. Results are not yet available (funded by Shire; NCT 02584959) ^41^.

Lanadelumab (DX-2930) is a new prophylactic agent that is an anti-kallikrein monoclonal antibody that inhibits kallikrein activity. In a phase 1b study, lanadelumab administered at a dose of 300 mg or 400 mg subcutaneously reduced cleavage of high-molecular-weight kininogen in plasma in patients with HAE to levels approaching that of individuals without the disorder. From day 8 to day 50, the 300 mg and 400 mg groups had 100% and 88% fewer attacks, respectively, than did the placebo group. Early data from the phase 3 study also suggests that twice-monthly therapy is very effective (funded by Shire; NCT02093923) ^42^.

APeX-1 is a two-part, phase 1b, randomized, double-blind, placebo-controlled dose-ranging trial to evaluate the safety, tolerability, pharmacokinetics, pharmacodynamics, and efficacy of orally administered once daily (QD) BCX7353 350 mg (kallikrein inhibitor) for 28 days as a prophylactic therapy. The results of this interim analysis are encouraging from the first part, which was recently released ^43^. Analysis of peripheral and abdominal attacks showed reductions of 88% and 24%, respectively, for BCX7353 compared with placebo. The failure to suppress abdominal attacks appeared to be that patients interpreted adverse effects of the medication as HAE attacks. One patient who had pre-existing colitis, hepatic steatosis, and over 20 years of previous androgen use exhibited an alanine aminotransferase (ALT) elevation of over three times the upper limit of normal at the end of BCX7353 treatment that later resolved. Side effects like diarrhea and flatulence were reported at current dose. The APeX-1 trial has been amended to add a 62.5 mg QD dose level and to increase the number of subjects at the 125 mg QD and 250 mg QD dose levels in order to more fully characterize dose response. The study is expected to be completed in December 2017 (funded by BioCryst Pharmaceuticals; NCT02870972) ^43, 44^. It is currently being studied in adults but will certainly be a welcome addition in children, since it is orally administered.

A phase 2 multicenter, randomized, double-blind, placebo-controlled, three-period crossover study to evaluate the efficacy and safety of recombinant human C1-Inh in the prophylaxis of angioedema attacks in patients with HAE has also been undertaken. A dose of 50 IU/kg was given intravenously twice weekly and reduced the attack frequency by 72% (95% CI: 63–81) and once-weekly Ruconest treatment reduced attack frequency by 44% (95% CI: 27–62) as compared with placebo. Adverse effects were minimal (funded by Pharming Technologies B.V.; NCT02247739) ^45^.

The bradykinin receptor antagonist icatibant (Firazyr), which is approved to treat attacks in adults, is being evaluated for the treatment of attacks in children and will be a welcome addition, since it is subcutaneously administered (funded by Shire; NCT01386658) ^46^.

## Conclusion

While symptoms may start in early childhood, attacks are typically infrequent in pre-pubertal children. Early onset of symptoms and frequent attacks in childhood predict more severe disease in adulthood ^7, 47^. Both boys and girls tend to experience more attacks, as well as more severe attacks, around puberty. It is unclear why this occurs in boys, but worsening in girls may be caused by increased levels of endogenous estrogen. The distribution of attacks in adolescent patients typically mirrors that in adults ^28^.

As more therapies for HAE become available, post-marketing surveillance becomes all the more important to assess for effectiveness and adverse effects of treatments because adverse events may be difficult to determine based on one or two phase 3 studies with a limited number of patients and treatment ^48^.

Because of the impact that HAE has on quality of life, productivity, anxiety, and absenteeism, prophylactic agents that have efficacy approaching 90% are critical. We anticipate that the new therapies under research will bring a revolution to the disease not only because of the efficacy but also because of the ease of administration and minimal toxicity of these medications. Unfortunately, only the CSL C1-Inh for subcutaneous use (CSL 830) and the intravenous use of Ruconest are being studied as a weight-adjusted dose, and the authors believe that dosing children, especially very small children, should not be similar to doses equal to that of an overweight adult.

As the field of HAE advances, work has been done on discovering prognostic and predictive biomarkers. In a GEE linear regression model, the presence of F12-46C/T was significantly associated with a 7-year delay in disease onset ( *P*<0.0001) regardless of *SERPING1* mutational status. It is concluded that F12-46C/T carriage acts as an independent modifier of C1-Inh-HAE severity ^49^. A similar relationship with the *KLKB1* gene has been noted but not yet published. We anticipate that, in the future, biomarkers will be used to determine those at risk of attacks early in life and those who may benefit from early initiation of prophylactic therapy.

C1-Inh-HAE represents one in five patients presenting with kinin-dependent angioedema, the great majority associated with heredity, and identified as nC1-Inh-HAE ^50^. C1-Inh-HAE could be considered as a prototypical condition for extending clinical trials to patient groups identified by ongoing and foreseen biomarkers and susceptibility genes. This will meet the expectations of many young patients and clinicians.

## Summary

Present therapies available for children are directed towards the treatment of attacks, and Cinryze is only 50% effective for prophylaxis. Androgens have dangerous adverse events for children and for this reason are not desirable. There is definitely a need for better prophylactic therapies, and the studies mentioned above look promising. With what appears to be well-tolerated subcutaneous or even oral medication that suppresses attacks by about 90%, we expect a major shift in how we treat HAE, even in children, in that most patients will be placed on prophylaxis.
